# Implications of Professional Occupation Related to Obesity in Patients Undergoing Bariatric Surgery

**DOI:** 10.3390/ijerph17155557

**Published:** 2020-07-31

**Authors:** José-María Jiménez, Sara García, Miguel-Ángel Carbajo, María López, María-José Cao, Jaime Ruiz-Tovar, María-José Castro

**Affiliations:** 1 Nursing Faculty, University of Valladolid, 47005 Valladolid, Spain; josejimenez@enf.uva.es (J.-M.J.); maria.lopez.vallecillo@uva.es (M.L.); mjcao@enf.uva.es (M.-J.C.); mariajose.castro@uva.es (M.-J.C.); 2 Centre of Excellence for the Study and Treatment of Obesity and Diabetes, 47005 Valladolid, Spain; doctormacarbajo@gmail.com (M.-A.C.); jruiztovar@gmail.com (J.R.-T.)

**Keywords:** obesity, bariatric surgery, weight loss, occupations, professional fields

## Abstract

Obesity is an epidemic with severe consequences on the professional development of patients. Bariatric surgery has proven to be a safe treatment with effective results in weight control. The aim of this study is to assess the implications of professional occupation in relation to the development of obesity and weight changes after bariatric surgery. We analyzed 500 obese patients (77.8% women, 22.2% men) who underwent one anastomosis gastric bypass surgery at the Centre of Excellence for the Study and Treatment of Obesity and Diabetes (2014–2019), assessing the influence of professional occupation on body composition and evolution of weight loss up to two years after surgery. Preoperative obesity type III and IV was higher in men than in women (45.9–19.8% vs. 43.7–9.5%; respectively). Prevalent clinical history in women was depression (46.7%), varicose veins (35.6%), and thyroid disease (9.7%), while in men it was respiratory failure (98.2%), high blood pressure (56.8%), hepatic steatosis (82%). Postoperative weight loss was effective in every professional field, reaching normal weight values from 12 months after surgery.

## 1. Introduction

Obesity is a worldwide epidemic that represents one of the most significant public health concerns globally [1,2]. The tendency of the prevalence of overweight and obesity at a global level has increased over the years, registering in 2016 more than 650 million obese people. Approximately 39% of the adult population were overweight (40% of women and 39% of men) and 18% were obese (15% of women and 11% of men) [1,2,3].

Direct costs caused by obesity represent about 9% of total healthcare expenses in developed countries, where overweight and obesity are more frequent [1,4]. Furthermore, obesity is associated with other kind of expenses that result in indirect costs, including reduced professional performance and productivity, as well as absenteeism and disability related to injuries and comorbidities [5,6]. In fact, the connection between obesity and work similarly affects workers who see their employment opportunities limited, leading to lack of motivation and psychological problems such as depression, leading to deterioration with both physical and psychological consequences [7,8]. 

Some articles describing obesity in relation to professional field, display how it is more frequent in sedentary jobs [9], which causes difficulties work performance, but they do not provide the figures in all productive sectors [10]. Some companies have made efforts to implement programs that encourage their workers to take better care of themselves and their weight. This translates into improved efficiency and workplace integration [11].

Bariatric surgery has proven to be an effective long-term treatment in the weight control and comorbidities associated with obesity [5,12,13]. Multiple studies show improvements in work performance, decreased absenteeism and increased productivity [14,15]. Even a rise in employability has been reported in those who have undergone bariatric surgery [16].

There are different surgical techniques of restrictive and malabsorptive type; the criteria of choice depend on each patient and the surgical team. The International Federation for the Surgery of Obesity and Metabolic Disorders (IFSO) has described bariatric surgeries worldwide in order to establish a profile of surgery patients in the future [17]. Among the malabsorptive surgeries, the one anastomosis gastric bypass (OAGB) stands out, as it is minimally invasive with effective long-term results in weight loss and associated comorbidities [18].

The assessment of body composition and anthropometric changes has been evaluated for the different types of surgical techniques, and malabsorptive type surgeries obtain better long-term results [16]. However, there are no published studies that analyze the professional field of surgery patients and display their results in weight changes [19]. The aim of this study is to describe the profile of the bariatric surgery prospective patient and the role of the professional occupation field in postoperative weight control changes.

## 2. Materials and Methods 

### 2.1. Study Design and Participants

Cross-sectional descriptive study conducted on 500 obese patients undergoing one anastomosis gastric bypass (OAGB) bariatric surgery at the Centre of Excellence for the Study and Treatment of Obesity and Diabetes, Valladolid (Spain) from January 2014 to July 2019. The inclusion criteria of the patients in the study followed the recommendations approved by the International Federation for the Surgery of Obesity and Metabolic Disorders (IFSO): BMI > 40 kg/m^2^, or BMI > 30–35 kg/m^2^ with the existence of associated comorbidities and poor metabolic control, of legal age and willing to participate in the study by giving their written consent [20].

### 2.2. Clinical Assesment and Data Collection

The preoperative protocol included both the selection criteria for bariatric surgery and the nutritional, psychological, blood, radiological, and cardiorespiratory function assessment, as well as other complementary tests in relation to existing comorbidities depending on the type of patient. This protocol was conducted by a multidisciplinary team following the recommendations in management and collection of clinical parameters described by the IFSO [21].

The clinical assessment of patients was conducted from the first preoperative consultation in which compliance with the selection criteria for surgery prospective patients was evaluated. If a patient met the criteria, he or she was included in the individualized preoperative protocol based on preoperative nutritional monitoring to enhance the commitment to postoperative dietary changes as well as to increase the safety of the surgical procedure.

The initial assessment included the collection of clinical background and socio-demographic data to complete the initial medical history of each patient according to their personal characteristics and specificities. In the consultation prior to surgery, the following were evaluated: age, gender, height, clinical and surgical history, professional occupation (Organization of Ibero-American States for Education, Science and Culture Classification. Annex 1) [22], body composition and anthropometrics (weight, BMI), and weight loss expression criteria. Anthropometric and weight loss changes were assessed in postoperative controls: 3, 12, 18, and 24 months after surgery.

Body Mass Index (BMI):

BMI = [weight(kg)/height^2^(m)] (1)

Weight loss expression criteria:

Percentage of Excess of Body Mass Index Loss (EBMIL):

% EBMIL = [(Preoperative BMI—Current BMI)/(Preoperative BMI–25)] × 100(2)

Percentage of Excess Weight Loss (EWL):

% EWL = [(Preoperative weight—Current weight)/(Preoperative weight—Ideal weight*)] × 100(3)

*The ideal body weight was determined according to Metropolitan Life Insurance Company’s formula.

Ideal weight = 50 + [0.75 × (Height (cm)—150)](4)

### 2.3. Surgical Procedure

OAGB bariatric surgery has been described in previous publications [23,24]. It was performed in every patient by the same surgical team, via laparoscopic access and under general anesthesia. This surgical technique involves terminolateral gastrojejunal anastomosis with an excluded biliopancreatic loop length calculated for each patient based on age, gender, degree and progression of obesity in order to improve weight control success and avoid nutritional complications [25].

### 2.4. Ethical Considerations

The study was conducted in accordance with the recommendations of the 1964 Declaration of Helsinki (last amendment, 2013). It has been approved by the Research Commission of the Nursing School of Valladolid (Ethical approval number: 2019/JMJP63). The informed consent was received from all the participants in the study and the data treatment was anonymous and confidential. There is no conflict of interest and no funding from public or private institutions.

### 2.5. Statistical Analysis

We created an Excel database (Microsoft Office 2016) to gather data. The data were recorded by one person and, after review, they were analyzed using the statistical program IBM SPSS Statistics v. 25.0.

Quantitative variables were described as mean ± standard deviation and their normalization was established with the Kolmogorov-Smirnov test. Qualitative variables were described via absolute and relative frequencies (percentages). To study the association between qualitative variables, the Chi-square test was used with Fisher’s exact test or likelihood ratio, depending on the conditions of application. To study the differences between mean values, the Student *t* test or Mann-Whitney U test were applied, depending on the conditions of application, for two groups; and the ANOVA or Kruskal-Wallis H test, also depending on the conditions of application, for more than two groups. The significance level for all tests was set at *p* ≤ 0.05.

## 3. Results

The study sample are 500 patients, average age of 40.8 ± 11.6 years, 389 women (77.8%) and 111 men (22.2%). There were no significant age differences between women (40.7 ± 11.7 years) and men (41.5 ± 11.2 years).

**Figure 1:**
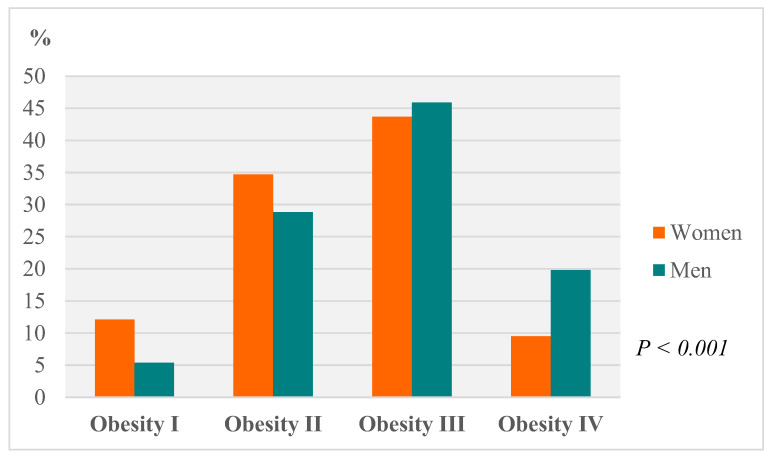
Distribution of patients by classification criteria according to BMI and gender at initial consultation (Obesity I = BMI 30–34.9; Obesity II = BMI 35–39.9; Obesity III = BMI 40–49.9; Obesity IV = BMI > 50).

Patient distribution based on the degree of overweight and obesity [26] is detailed in Figure 1, with a higher proportion of women suffering type I and type II obesity, as opposed to men, who had a higher prevalence of type III and type IV obesity (*p* < 0.05).

In the anamnesis, clinical history was assessed, and we found respiratory failure, arthropathies, obstructive sleep apnea syndrome, and hepatic steatosis to be the most prevalent conditions. The clinical history described in Table 1 shows that women suffered from a higher rate of varicose vein disease than men (35.6% vs. 21.6%. *p* < 0.05), thyroid disease (9.7% vs. 3.6%. *p* < 0.05), depression (46.7% vs. 18.9%. *p* < 0.05), and were more under psychiatric treatment (23.8% vs. 9.9% *p* < 0.05).

In contrast, high blood pressure (56.8% vs. 24.4%. *p* < 0.05), treatment with oral antihypertensive drugs (36.9% vs. 16.2%. *p* < 0.05), obstructive sleep apnea syndrome (86.5% vs. 77.7%. *p* < 0.05), respiratory failure (98.2% vs. 93.6%. *p* < 0.05), hepatic steatosis (82% vs. 67.7%. *p* < 0.05), and the presence of hiatal hernia (56.8% vs. 46.7%. *p* < 0.05) prevailed in men. 

Regarding the surgical history of the patients, trauma surgery (14.4%) was the most common, followed by appendectomy (10.8%). Women had a higher frequency of previous surgeries than men in the case of cholecystectomy (7.7% vs. 0.9%. *p* < 0.05). 11.5% of women had previously carried a gastric balloon compared to 3.6% of the men, and 7.2% had undergone liposuction compared to 0.9% of the men (*p* < 0.05). 

**Figure 2:**
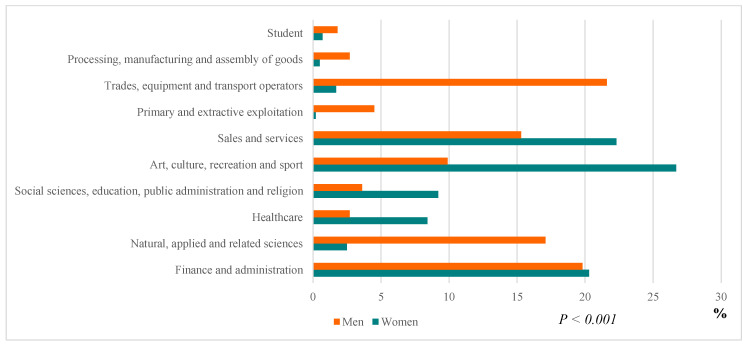
Classification of patients by occupation and gender.

The professional occupation of patients was distributed in nine major fields [22], adding the category “student.” Most patients work in arts, culture, recreation and sports (23%), sales and services (20.8%), and finance and administration (20.2%). Women’s professional occupations surpass men’s in the arts, culture, recreation and sport field (26.7% vs. 9.9%. *p* < 0.05), sales and services (22.3% vs. 15.3%. *p* < 0.05), and healthcare (8.4% vs. 2.7%. *p* < 0.05). In contrast, men’s professional occupations are higher in the trade, equipment and transport operators (21.6% vs. 1.7%. *p* < 0.05), applied and related natural sciences (17.1% vs. 2.5%. *p* < 0.05), primary and extractive exploitation (4.5% vs. 0.2%. *p* < 0.05), and in processing, manufacturing, and assembly of goods (2.7% vs. 0.5%. *p* < 0.05) Figure 2.

Postoperative changes in weight loss were analyzed according to the type of professional occupation (Table 2). The highest average weight before surgery was found in students (136.1 ± 24.3 kg), followed by the primary and extractive exploitation field occupations (129.7 ± 37.7 kg), *p* < 0.05. Regardless of preoperative weight, the trend in all patients in all professional fields covered was a significant decrease in weight and BMI.

There are variabilities among professional fields regarding the point of lowest average weight during the first 24 postoperative months. In the finance and administration field, this was reached at 18 months (66.2 ± 14.1); at 12 months in natural, applied, and related sciences (78.4 ± 15.3 kg), healthcare (66.4 ± 11.9 kg), social sciences, education, public administration, and religion (66.7 ± 11.7 kg), primary and extractive exploitation (65 ± 1.4 kg), trades, equipment operators and transport fields (76.3 ± 13.2 kg); and at 24 months in the arts, culture, recreation and sport (65.4 ± 10.1 kg), sales and services (64.8 ± 9.1 kg), processing, manufacturing and assembly of goods fields (70.5 ± 8.1 kg), and students (74.3 ± 11.9 kg).

Weight loss expressed in relative terms like % EWL and % EBMIL shows the highest weight loss at 24 months in the processing, manufacturing, and assembly of goods field (99.2 ± 8.3%, 129.3 ± 13.2% respectively). In contrast, the primary and extractive exploitation field had the lowest weight loss in relative terms at 18 months: % EWL (78.5 ± 15.5%) and % EBMIL (93.8 ± 18.3%).

In the professional fields where there were more women than men (arts, culture, recreation and sport; sales and services and healthcare), men were found to have a higher average initial weight in every field: 136.6 ± 19.9 kg vs. 109.5 ± 18.1 kg; 129.3 ± 24.1 kg vs. 108.19 ± 16.87 kg; 142 ± 9 kg vs. 111.7 ± 20.7 kg respectively. *p* < 0.05.

**Figure 3:**
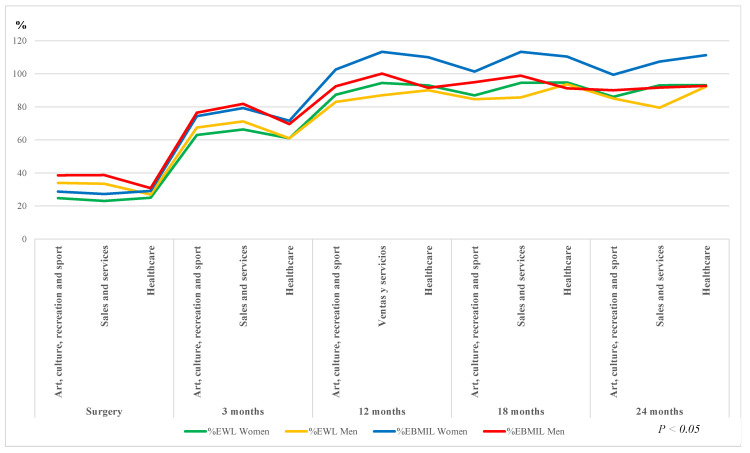
Evolution of related weight variations by professional occupation and gender. Measurement units: % excess weight loss (EWL) and % excess of body mass index loss (EBMIL).

Nevertheless, the trend in these three professional areas was similar, with significant weight loss, expressed in % EWL and % EBMIL, in all the controls carried out since the preoperative and in the following ones, as illustrated in Figure 3.

## 4. Discussion

Bariatric OAGB surgery has enabled effective weight control in the patients studied, illustrating which professional fields have a greater impact on weight control. Our study revealed some of the main professional areas associated with the obesity of their workers, highlighting a greater presence of women in areas such as art and culture, sales and services, and the healthcare system. The profile of obese patients in different professional fields is highly variable and responds to different causes [10,27]. There is no evidence of studies that directly analyzes the impact of bariatric surgery and its evolution depending on the type of professional activity [19], but studies such as the US National Health Interview Survey [10] describe a greater proportion of obesity in women in areas such as finance and administration and healthcare, as opposed to areas such as construction, assembly and transport where men are more likely to be obese.

Psychiatric disorders and psychological problems are clinical determining factors in the development of obesity and are correlated with emotional state and professional performance [7,8]. Almost half of the women in our study (46.7%) experienced depression and only 23.8% were under treatment, numbers that are slightly higher than those reported in other studies of bariatric surgery candidates [28].

The evolution of weight loss follows a similar trend to that observed in several reports of patients undergoing malabsorptive surgery [29]. In our study, average weight normalization was observed in every professional field described from 12 months after surgery, with similar results to other groups of OAGB patients up to 24 months [23,25]. The % EBMIL was significantly higher in women from the 12th postoperative month, although the mean initial weight of men was higher in every professional field.

Patients with a greater degree of obesity are correlated with a higher rate of unproductive work, especially in the trades, equipment operators, and transport sectors [10]. In our study, workers on this field were mainly men and achieved significant weight loss results, although men working in other fields such as primary and extractive industries, and natural, applied, and related sciences were found to have greater weight loss with a similar average initial weight.

Bariatric surgery has proven to be an effective long-term treatment in the control of obesity and associated comorbidities [13,30]. Previous studies [11,16,19,31] have reported improved work performance and employability of patients undergoing bariatric surgery, demonstrating a positive correlation between weight loss and improved work productivity regardless of the professional field, as well as a reduction in direct and indirect costs for the companies [4,5,6].

Among the limitations of this study is the lack of randomness of the selected sample, since all patients belong to the same care center where the OAGB was performed. Although the objective of the study was to analyze the results in the first 24 months after surgery, it is necessary to continue with the follow-up process of the patients analyzed in the current study and to assess the long-term impact of the results on weight control and associated comorbidities by comparing the different professional fields.

## 5. Conclusions

This study describes the profile of the patient undergoing bariatric surgery, its socio-demographic aspects, and the evolution in weight control at 24 months after OAGB surgery. Patients’ professional occupation was a key factor in obesity and its outcome after surgery. However, it is necessary to assess the evolution of each patient individually, considering aspects such as physical activity required for each job, and dietary and lifestyle changes. In all the professional fields analyzed, average normal weight values were reached (BMI 18.5–24.9 kg/m2) from 12 months after surgery. Women predominated in professional fields such as art and culture, sales and services, and healthcare, and they presented a greater weight loss than men.

## Figures and Tables

**Table 1:** Clinical history of the patients by gender.

	% of Men (n)	% of Men (n)	Total % (n)
**High blood pressure**	24.4% (95)	56.8% (63) *	31.5% (158)
**Oral anti-hypertensive drugs treatment**	16.2% (63)	36.9% (41) *	20.8% (104)
**Diabetes Mellitus**	13.1% (51)	18% (20)	14.2% (71)
**Treatment with oral anti-diabetics**	6.2% (24)	7.2% (8)	6.4% (32)
**Insulin treatment**	1% (4)	3.6% (4)	1.6% (8)
**Obstructive sleep apnea syndrome**	77.7% (303)	86.5% (96) *	79.6% (399)
**Respiratory failure**	93.6% (365)	98.2% (109) *	94.6% (474)
**Arthropathy**	84.6% (330)	78.4% (87)	83.2% (417)
**Varicose veins**	35.6% (139) *	21.6% (24)	32.5% (163)
**Dyslipemia**	56.9% (222)	64.9% (72)	58.7% (294)
**Hepatic steatosis**	67.7% (264)	82% (91) *	70.9% (355)
**Gastroesophageal reflux**	35.4% (138)	37.8% (42)	35.9% (180)
**Hiatal hernia**	46.7% (182)	56.8% (63) *	48.9% (245)
**Cholelithiasis**	13.1% (51)	8.1% (9)	12% (60)
**Thyroid disease**	9.7% (38) *	3.6% (4)	8.4% (42)
**Anxiety**	1.8% (7)	-	1.4% (7)
**Depression**	46.7% (182) *	18.9% (21)	40.5% (203)
**Psychiatric treatment**	23.8% (93) *	9.9% (11)	20.8% (104)

* *p* < 0.05 when comparing results by gender.

**Table 2:** Evolution of weight loss in the different professional fields at the time of the postoperative follow-up controls.

		Preoperative	3 Months	12 Months	18 Months	24 Months
Finance and administration	Weight	110.6 ± 20	78.3 ± 12.6 *	68.4 ± 13.1 *	66.2 ± 14.1 *	68.2 ± 15.1 *
	BMI	40.8 ± 5.7	29 ± 3.8 *	25 ± 3.5 *	24.2 ± 3.3 *	25 ± 3.7 *
	% EWL	24.9 ± 9.5	66.8 ± 13.8 *	90.2 ± 19 *	93.3 ± 17.9 *	89.3 ± 19.2 *
	% EBMIL	28.7 ± 11	79.9 ± 21.4 *	106 ± 29.8 *	111.3 ± 27.4 *	107.4 ± 30 *
Natural, applied and related sciences	Weight	126.9 ± 29	86.1 ± 15.6 *	78.4 ± 15.3 *	82.7 ± 13.5 *	84.8 ± 7.6 *
	BMI	43.3 ± 8.2	29.5 ± 4.9 *	26.2 ± 3.7 *	26.7 ± 2.7 *	27.1 ± 1.9 *
	% EWL	30.6 ± 11.1	69.7 ± 14.8 *	84.9 ± 14 *	82.2 ± 11.2 *	80.6 ± 4.8 *
	% EBMIL	37.1 ± 14	82.6 ± 16.2 *	98.1 ± 19.3 *	92.1 ± 12 *	91.7 ± 8.4 *
Healthcare	Weight	114.3 ± 21.6	81.9 ± 14.1 *	66.4 ± 11.9 *	70.1 ± 6.9 *	66.6 ± 9 *
	BMI	41.9 ± 6.3	29.9 ± 3.5 *	24.1 ± 3.5 *	23.9 ± 2.3 *	23.5 ± 3.2 *
	% EWL	25.1 ± 7.3	61 ± 13.3 *	92.9 ± 13.9 *	94.8 ± 9.8 *	93.1 ± 16.6 *
	% EBMIL	29.3 ± 8.3	71.3 ± 16.1 *	110.1 ± 18.4 *	110.4 ± 15.4 *	111.3 ± 20.3 *
Social sciences, education, public administration and religion	Weight	114.3 ± 22.2	82.8 ± 17.6 *	66.7 ± 11.7*	67.6 ± 15.3 *	72.9 ± 18.2 *
	BMI	42.2 ± 6.1	30.4 ± 4.8 *	24.6 ± 3.5 *	25.6 ± 4.7 *	25 ± 5.3 *
	% EWL	2.8 ± 9	62.2 ± 14.5 *	90 ± 16 *	87.4 ± 21.9 *	96.2 ± 20.6 *
	% EBMIL	26.6 ± 9.9	73.1 ± 20.2 *	107 ± 21.7 *	104.3 ± 25.2 *	118.2 ± 20.9 *
Art, culture, recreation and sport	Weight	112.1 ± 19.8	79.5 ± 14.2 *	67.1 ± 15.3 *	66.2 ± 13.5 *	65.4 ± 10.1 *
	BMI	43 ± 6.7	30.7 ± 5.1 *	26.11 ± 5.4 *	25.6 ± 3.9 *	25.7 ± 3.2 *
	% EWL	25.6 ± 9.3	63.4 ± 15.7 *	87 ± 18.2 *	86.8 ± 16.1 *	86.1 ± 14.2 *
	% EBMIL	29.7 ± 10.8	74.6 ± 22.7 *	101.9 ± 23.4 *	100.9 ± 21.7 *	99.4 ± 20.1 *
Sales and services	Weight	111.6 ± 19.7	78.1 ± 13.1 *	65.4 ± 10.4 *	66.3 ± 10 *	64.8 ± 9.1 *
	BMI	41.5 ± 6.2	29.2 ± 4.6 *	24.3 ± 2.9 *	24.3 ± 2.5 *	24.6 ± 2.7 *
	% EWL	24.8 ± 9.9	67 ± 15.9 *	93 ± 15.2 *	92.2 ± 13.8 *	90.5 ± 13.7 *
	% EBMIL	29.1 ± 11.4	79.7 ± 22.6 *	110.7 ± 24.7 *	109.6 ± 24.3 *	104.5 ± 17.9 *
Primary and extractive exploitation	Weight	129.7 ± 37.7	79.2 ± 7.7 *	65 ± 1.4 *	72.5 ± 7.7 *	69.8 ± 7 *
	BMI	44.8 ± 13.1	27.5 ± 3.2 *	25.4 ± 2.3 *	25.8 ± 2.4 *	25.2 ± 1.8 *
	% EWL	30.6 ± 4.4	74.1 ± 14.5 *	82.6 ± 17.6 *	78.5 ± 15.5 *	84 ± 13*
	% EBMIL	35.7 ± 4.1	86.3 ± 18.2 *	94.4 ± 14.8 *	93.8 ± 18.3 *	97.5 ± 12.9 *
Trades, equipment and transport operators	Weight	125.7 ± 19.7	84.4 ± 11.4 *	76.3 ± 13.2 *	78.3 ± 10.9 *	81.2 ± 8.5 *
	BMI	42.3 ± 5.9	28.4 ± 3.9 *	25.3 ± 3.3 *	26.4 ± 3.8 *	25.8 ± 3.4 *
	% EWL	30.6 ± 9.9	70.5 ± 15.3 *	86 ± 14.8 *	79.1 ± 17.3 *	79.9 ± 15.2 *
	% EBMIL	35.9 ± 12	83.5 ± 21 *	101.2 ± 19.1 *	94.5 ± 23.7 *	97.2 ± 19.9 *
Processing, manufacturing and assembly of goods	Weight	124.1 ± 21.9	85.6 ± 9.9 *	71.6 ± 9.7 *	72.4 ± 13.8 *	70.5 ± 8.1 *
	BMI	38.7 ± 4.8	27.9 ± 2 *	23.2 ± 0.9 *	23.6 ± 1.7 *	22.5 ± 1.4 *
	% EWL	30.8 ± 7.6	67.5 ± 8.8 *	95.2 ± 4.2 *	94.4 ± 7.7 *	99.2 ± 8.3 *
	% EBMIL	37 ± 7.4	81.3 ± 14.1 *	114.3 ± 9.2 *	110.2 ± 11.8 *	129.3 ± 13.2
Student	Weight	136.1 ± 24.3	89.6 ± 11.1 *	74.4 ± 9.9 *	75.1 ± 12.5 *	74.3 ± 11.9 *
	BMI	46.9 ± 6.7	31 ± 3.1 *	25.7 ± 1.8 *	25.8 ± 2.1 *	25.5 ± 1.9 *
	% EWL	28.8 ± 5.5	64,9 ± 8.4 *	87.4 ± 5.5 *	86.9 ± 6 *	87.9 ± 5.5 *
	% EBMIL	32.3 ± 5.6	73 ± 9.5 *	98.5 ± 8.8 *	97.9 ± 9.3 *	99.1 ± 8.8 *

* *p* < 0.05 compared to preoperative control. Results were expressed with mean ± standard deviation. Units of measurement: Weight (kg), Body Mass Index (BMI) (kg/m^2^), Percentage of Excess Weight Loss (EWL) (%), Percentage of Excess of Body Mass Index Loss (EBMIL) (%).
